# Comparative immune responses of corals to stressors associated with offshore reef-based tourist platforms

**DOI:** 10.1093/conphys/cov032

**Published:** 2015-07-24

**Authors:** Jeroen A J M van de Water, Joleah B Lamb, Madeleine J H van Oppen, Bette L Willis, David G Bourne

**Affiliations:** af1 ARC Centre of Excellence for Coral Reef Studies, James Cook University, Townsville, QLD 4811, Australia; af2 College of Marine and Environmental Sciences, James Cook University, Townsville, QLD 4811, Australia; af3 AIMS@JCU, James Cook University, Townsville, QLD 4811, Australia; af4 Australian Institute of Marine Science, PMB 3, Townsville MC, Townsville, QLD 4810, Australia; af5 Department of Ecology and Evolutionary Biology, Cornell University, Ithaca, NY 14850, USA; af6 School of BioSciences, The University of Melbourne, Parkville, VIC 3010, Australia

**Keywords:** coral, disease, GFP-like proteins, immunity, phenoloxidase, tourism

## Abstract

Unravelling the contributions of local anthropogenic and seasonal environmental factors in suppressing the coral immune system is important for prioritizing management actions at reefs exposed to high levels of human activities. Here, we monitor health of the model coral *Acropora millepora* adjacent to a high-use and an unused reef-based tourist platform, plus a nearby control site without a platform, over 7 months spanning a typical austral summer. Comparisons of temporal patterns in a range of biochemical and genetic immune parameters (Toll-like receptor signalling pathway, lectin–complement system, prophenoloxidase-activating system and green fluorescent protein-like proteins) among healthy, injured and diseased corals revealed that corals exhibit a diverse array of immune responses to environmental and anthropogenic stressors. In healthy corals at the control site, expression of genes involved in the Toll-like receptor signalling pathway (*MAPK p38*, *MEKK1*, *cFos* and *ATF4/5*) and complement system (*C3* and *Bf*) was modulated by seasonal environmental factors in summer months. Corals at reef platform sites experienced additional stressors over the summer, as evidenced by increased expression of various immune genes, including *MAPK p38* and *MEKK1.* Despite increased expression of immune genes, signs of white syndromes were detected in 31% of study corals near tourist platforms in the warmest summer month. Evidence that colonies developing disease showed reduced expression of genes involved in the complement pathway prior to disease onset suggests that their immune systems may have been compromised. Responses to disease and physical damage primarily involved the melanization cascade and GFP-like proteins, and appeared to be sufficient for recovery when summer heat stress subsided. Overall, seasonal and anthropogenic factors may have interacted synergistically to overwhelm the immune systems of corals near reef platforms, leading to increased disease prevalence in summer at these sites.

## Teaser

We investigated the effect of stressors associated with offshore reef-based tourist platforms on the coral immune system. Our findings suggest that stressors associated with platform-related activities, in synergy with seasonal factors, compromise the coral immune system, contributing to the higher coral disease prevalence observed at these locations.

## Introduction

Increasing evidence that coral disease epizootics are causing significant declines in coral cover and degradation of coral reefs (Porter *et al.*, 2001; Gardner *et al.*, 2003; Osborne *et al.*, 2011) suggests that coral immune systems are being overwhelmed by a combination of both anthropogenic and naturally occurring environmental disturbances at local and global scales. Teasing apart the roles of climate change-related environmental factors, such as warming and acidifying oceans, in disease causation from other anthropogenic disturbances, such as sedimentation, sewage disposal or eutrophication caused by agricultural run-off (Bruno *et al.*, 2007; Harvell *et al.*, 2007; Lesser *et al.*, 2007; Haapkylä *et al.*, 2011; Sutherland *et al.*, 2011; Ruiz-Moreno *et al.*, 2012; Redding *et al.*, 2013; Lamb *et al*. 2014; Pollock *et al*. 2014), is key to understanding current challenges facing coral immune systems. A report of 15-fold greater prevalence of coral disease near permanent tourist reef platforms compared with adjacent reefs without such platforms (Lamb and Willis, 2011) suggests that such sites are ideal microcosms for characterizing responses of the coral innate immune system. With coral-reef based tourism becoming one of the fastest growing tourism sectors worldwide (Ong and Musa, 2011), determining whether differential coral immune responses can distinguish among anthropogenic and environmental drivers of reduced coral health would represent a significant step forward in the development of effective coral reef management and conservation strategies for the tourism industry.

Corals have a large repertoire of innate immune defence mechanisms available to maintain fitness and defend against biotic and abiotic stressors. Three of these have been relatively well documented, as described in further detail below: (i) the Toll-like receptor (TLR) pathway; (ii) the melanization cascade; and (iii) the complement system. However, the manner in which these immune mechanisms respond to different environmental and anthropogenic impacts is relatively unexplored.

Toll-like receptors are activated following the detection of microbial components (microbe-associated molecular patterns) and subsequently activate various signal transduction pathways [e.g. c-Jun N-terminal kinase (JNK), mitogen-activated protein kinase (MAPK) p38 and nuclear factor-κB (NF-κB) pathways], which regulate the expression of target genes involved in immunity and cell survival, thereby orchestrating the immune response. Recent molecular studies of the coral innate immune system have identified a large number of genes encoding TLRs and proteins involved in the downstream signalling pathways (Miller *et al.*, 2007; Shinzato *et al.*, 2011; Hamada *et al.*, 2013); however, functional studies of the TLR signalling pathways in corals are limited.

The melanization cascade, or prophenoloxidase (proPO)-activating system (Mydlarz *et al.*, 2008; Palmer *et al.*, 2008), is a rapidly induced mechanism activated in response to microbe-associated molecular patterns (Cerenius *et al.*, 2010). The capacity to activate this system within minutes, leading to the production of a hostile cellular environment and, ultimately, to the deposition of melanin that immobolizes microbes (Cerenius *et al*., 2010), suggests that this immune mechanism may be directed primarily at events requiring a rapid response, such as pathogen invasion and injury. Significant correlations between phenoloxidase (PO) activity levels and disease resistance in various invertebrates, including corals, plus evidence of the major role that the proPO-activating system plays in the disease response and wound-healing process (Palmer *et al.*, 2010, 2011a, c), corroborates this interpretation. Although the biological function of the melanization cascade in corals has been studied extensively, the impacts of stressors other than elevated seawater temperatures are still unknown.

The complement system is another effector mechanism involved in the direct elimination of invading microbes, primarily via promoting phagocytosis and inducing the formation of the membrane attack complex. Key components of the complement system, including complement C3, Factor B (Bf), lectins and mannose-binding lectin-associated serine protease, are present in many invertebrates (Mydlarz *et al.*, 2006; Cerenius *et al*., 2010). In corals, lectins and C3 have been implicated in the antibacterial and wounding response (Kvennefors *et al.*, 2008, 2010; Brown *et al.*, 2013), and membrane attack complex/perforin domain-containing genes have been identified (Miller *et al*., 2007). How this immune mechanism is affected by environmental and anthropogenic factors, however, remains to be elucidated in corals.

Elevated seawater temperatures are known to reduce immunocompetence, which is the ability of an organism to exhibit an immune response, in several coral species (Palmer *et al*., 2011a, b; Vidal-Dupiol *et al.*, 2014). In other invertebrate systems, changes in salinity and elevated levels of nutrients or pollutants are also known to reduce immune system function, resulting in increased disease-related mortality (Coteur *et al.*, 2001; Reid *et al.*, 2003; Cheng *et al.*, 2004a, b; Liu and Chen, 2004; Tseng and Chen, 2004; Danis *et al.*, 2006; Li *et al.*, 2010; Ellis *et al.*, 2011). Higher prevalence of coral disease on reefs near permanent offshore tourist platforms than at reefs without such facilities (Lamb and Willis, 2011) suggests that platform-associated stressors also affect coral immunocompetence and, potentially, coral-associated microbial communities. As healthy bacterial communities are essential to the functioning of the coral holobiont, playing important roles in nutrient cycling (Raina *et al.*, 2009; Lema *et al.*, 2012) and protection from pathogens (Ritchie, 2006; Shnit-Orland and Kushmaro, 2009; Teplitski and Ritchie, 2009; Alagely *et al.*, 2011), any changes in the structure of bacterial communities may signify that immune systems of corals living near tourist platforms are compromised.

In this study, we monitored colonies of the reef-building coral *Acropora millepora* near tourist platforms and control sites over a summer season to establish baseline levels of a suite of immune parameters in corals in undisturbed environments and to assess the effects of platforms on coral immunocompetence based on temporal patterns in immune protein levels and gene expression. The occurrence of both injury (associated with snorkelling and diving activities) and disease at platform sites, coupled with the recovery of injuries and lesions as warm summer temperatures subsided, enabled us to compare the functional responses of a range of immune parameters to both anthropogenic and environmental factors.

## Materials and methods

### Study site and sample collection

**Figure 1:**
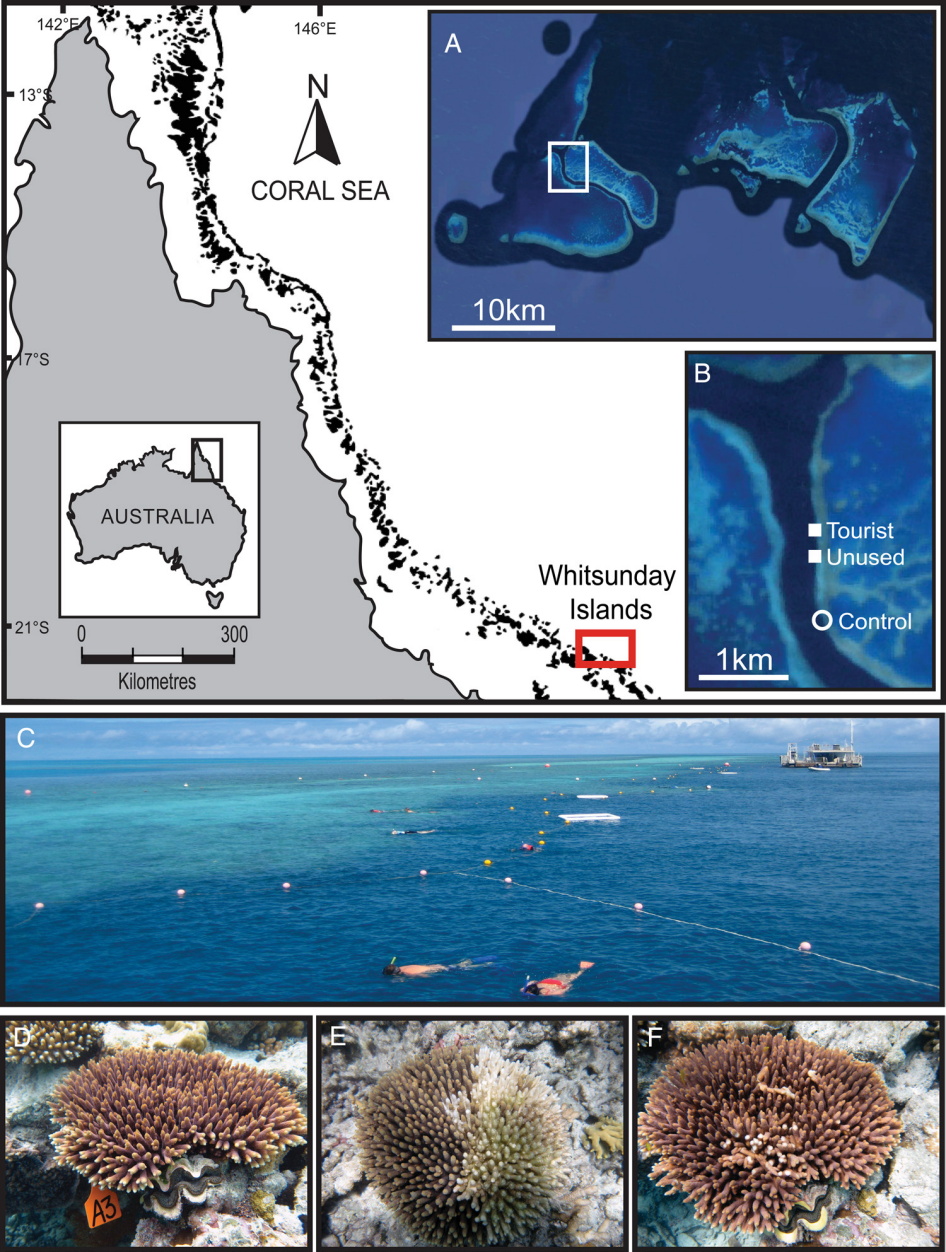
Maps showing the location of Hardy Reef (**A**), located 75 km offshore within the central sector of the Great Barrier Reef, Australia, and three study sites (**B**): a high-use visitor platform, an unused visitor platform and a control site with no platform. The unused platform is situated ∼300 m south of the high-use platform (**C**), and the control site lies an additional 800 m south of the unused platform. Photographs showing representative images of healthy (**D**), diseased (white syndrome; **E**) and damaged colonies (**F**) of the coral *Acropora millepora* in January 2011.

The study was conducted at Hardy Reef (19°44′33″S, 149°10′57″E) on the Great Barrier Reef of Australia, where two offshore platforms are permanently moored between November 2010 and June 2011 (Fig. 1A). The first platform (45 m × 12 m) was in use at the time of the study as the primary vessel mooring pontoon and could accommodate up to 400 visitors and associated recreational activities per day (study site 1; tourist platform), although such usage is significantly more than the recommended carrying capacity of 5000 recreational users per year at a single reef site (Hawkins and Roberts, 1997). A second platform (24 m × 10 m), which had not been used by tourists for a year prior to the study, was located 400 m south of the main tourist platform (study site 2; unused platform) (Fig. 1B and C). The platforms are located 5 m from the reef crest, and large numbers of seabirds rest on both platforms throughout the year. In addition, a control site (study site 3) was established 800 m south of the unused platform in similar reef habitat (Fig. 1B).

Eight visually healthy colonies of similar size of *A. millepora*, a model coral species widely used in physiological and genomic studies, were tagged using plastic cattle tags and cable ties at 2–3 m depth at each of the three study sites. Colonies were sampled at the following six time points: November (late austral spring), December (early austral summer), January (austral summer), February (austral summer), March (late austral summer) and June (early austral winter). At each sampling time point, the health status of the tagged coral colonies was visually assessed and categorized as healthy, damaged (branches recently broken, with exposed skeleton) or diseased (signs of the coral disease white syndrome, as per Beeden *et al.* 2008; Fig. 1D–F). One branch (∼5 cm in length) was sampled from midway between the centre and the edge of each tagged colony, at each of the six time points during the study. For diseased and damaged colonies, an apparently healthy portion of each branch was sampled ∼1 cm from the lesion boundary or damaged area. In all instances, the disease lesion was radiating from the centre of the colony. A photograph of each tagged colony was taken before and after each sample was collected. Samples were collected in the same order for all time points, and the sampling took ∼2 h to complete. Samples were immediately snap-frozen in liquid nitrogen and stored at −80°C.

### Messenger RNA isolation

Frozen samples of *A. millepora* were crushed in a liquid nitrogen-chilled, stainless-steel mortar and pestle using a hydraulic press. Messenger RNA was isolated from ∼100 mg of crushed coral using the Dynabeads mRNA DIRECT kit (Invitrogen Dynal AS, Oslo, Norway) according to a modified protocol based on the manufacturer’s recommendations (van de Water *et al*., 2015a). In short, crushed coral was added to 400 µl lysis buffer, incubated on a vortex at low speed for 7 min and centrifuged for 2 min at 12 000***g***. Supernatant was added to prewashed oligo(dT)-Dynabeads and incubated on the vortex at medium speed for 8 min to allow mRNA annealing. Tubes were placed on a DynaMag-2 magnetic particle concentrator for 5 min, and supernatant was removed. Using the DynaMag-2, oligo(dT) Dynabead–mRNA complexes were washed twice with 300 µl of Buffer A and subsequently twice with 400 µl of Buffer B. Complexes were resuspended in 27 µl ice-cold 10 mM Tris–HCl, incubated at 80°C for 2 min and rapidly cooled down on ice. Oligo(dT)-Dynabeads were concentrated on the DynaMag-2, and mRNA-containing supernatant was collected and stored at −80°C until use.

### Gene expression analysis

Expression levels of 17 immune system-related genes and four reference genes (Seneca *et al.*, 2010; Siboni *et al.*, 2012) were analysed using the GenomeLab GeXP Start Kit and the CEQ-8800 Genetic Analysis System (Beckman-Coulter, Brea, CA, USA) following the protocol described by Siboni *et al*. (2012) and van de Water *et al*. (2015a) with some modifications. A description of all genes and primer sequences can be found in Supplementary Table S1. For each sample, cDNA was generated from 6.7 ng of mRNA. Forward primer concentrations were 200 nM. Reverse primer concentrations were optimized for the multiplex to ensure that signals in the electropherogram were within the CEQ-8800 detection range, as follows: 2 µM for *CTL2*; 1 µM for *MEKK1*, *GAPDH*, *Bf*, *MAPK p38*, *ctg_1913*, *RPL9*, *CTL1*, *HL1* and *Apextrin*; 500 nM for *TRAF6*, *TIR-1*, *C3/A2M-2*, *ERK-2*, *Millectin*, *HL2* and *cJun*; 62.5 nM for *ATF4/*5; 25 nM for *cFos*; 12.5 nM for *NFκB*; and 23 pM for *RPS7*. The PCR products were diluted 1:20 prior to loading on the CEQ-8800 Genetic Analysis System. Data were filtered and analysed using the GeXP Fragment Analysis and eXpress Profiler software packages (Beckman-Coulter). Gene expression levels were normalized to an internal control (Kan^R^) and to the geometric mean of the expression levels of the three most stable reference genes (*RPS7*, *RPL9* and *ctg_1913*) selected using geNorm (Vandesompele *et al.*, 2002). Results were obtained for three independent technical replicates per sample.

### Phenoloxidase activity

Phenoloxidase activity was assayed according to procedures outlined by Palmer *et al*. (2011a), with some modifications. Both total potential (trypsin-activated) phenoloxidase (tpPO; van de Water *et al*., 2015b) activity and PO activity were measured to analyse the total capacity and the active fraction of the proPO system, respectively, in each sample. To analyse tpPO activity, 20 µl of coral tissue lysate was loaded in triplicate into wells of a 96-well plate, to which Tris-buffered saline (50 mM, pH 7.8; 40 µl) and trypsin (25 µl 0.1 mg/ml) were added. Reaction mixtures were incubated for 20 min to allow for activation of proPO by trypsin, and then 30 µl of 10 mM dopamine hydrochloride (Sigma-Aldrich, St Louis, MO, USA) was added to each mixture. As a blank, 20 µl of extraction buffer was used. The same procedure was followed to analyse PO activity, except that 25 µl double distilled water was substituted for the trypsin solution. Absorbance was measured at 490 nm every 5 min for 45 min using the SpectraMax M2 (Molecular Devices, Sunnyvale, CA, USA). Data for each sample were independently obtained in triplicate. Phenoloxidase activity was calculated as the change in absorbance using the linear portion of the reaction curve over time and standardized to the total protein content of each sample.

### Chromoprotein and fluorescent protein expression

Twenty microlitres of tissue lysate was added to each well of a black, clear-bottomed 384-well plate in triplicate for each sample. Expression of chromoprotein (CP) was analysed by measuring the absorbance at 588 nm using a SpectraMax M2. The fluorescence spectrum was analysed by measuring emission wavelengths between 400 and 700 nm, with a 5 nm resolution, emitted upon excitation of fluorescent proteins (FPs) at 280 nm. All data were normalized to total protein content. Fluorescence spectra and fluorescent protein expression levels were calculated in R using the method described by van de Water *et al.* (2015b). In summary, the exponentially decaying background scatter was subtracted from each spectrum between 445 and 645 nm, and multiple regression models based on purified FP spectra were fitted to the data to calculate the proportions of the individual fluorescent proteins [cyan (CFP), green (GFP) and red fluorescent protein (RFP)] present.

### Environmental parameters

Daily water temperature, rainfall accumulation, light intensity and wind speed data were collected by the Australian Institute of Marine Science (AIMS) monitoring station located at the main tourist platform (data available from http://data.aims.gov.au/aimsrtds/datatool.xhtml). The means for each environmental parameter were calculated using daily values from a 14 day period including and immediately preceding each month’s sampling date (Supplementary Fig. S1).

### Statistical analyses

Temporal patterns of individual gene and protein parameters in control site corals were analysed using a linear mixed effect model, with the random effect ‘colony’ as group variable, the response variable ‘immune parameter’ as a dependent variable, and time as an independent fixed effect. Outcomes were adjusted for multiple comparisons by a Tukey’s honest significant difference test. Analysis of variance (ANOVA) was used to test for differences in immune parameters between corals at the control site and corals with the following characteristics: (i) were healthy near the tourist platform; (ii) were healthy near the unused platform; (iii) sustained damage; and (iv) developed disease, followed by a Fisher’s least significant difference test. Because of low levels of gene expression resulting in a value of zero in a few cases, data sets with >20% zeros were tested for differences in the proportion of zero values across sites and health conditions using logistic regression models, and differences in expression of samples with expression values greater than zero between sites and health conditions using a linear mixed effect model. All analyses were conducted using the environment for statistical computing R (version 3.1.1). Data were considered significant when *P* < 0.05 or when the 95% confidence interval excluded zero.

## Results

### Coral health assessment

At the control site, all eight tagged colonies remained visually healthy and undamaged throughout the study period (from November to June; Fig. 1D). Colonies at the platform sites (i.e. the primary tourist platform and the unused platform) were visually healthy at the start of the study in November and December. In January, five colonies near the platforms (one at the tourist platform and four at the unused platform) developed macroscopic signs of white syndrome (WS), the collective name for a group of tissue-loss diseases (Willis *et al.*, 2004), and sustained ∼40–50% partial colony mortality (Fig. 1E). In addition, three of the tagged colonies at the tourist platform sustained severe physical damage, with many broken branches on each colony, consistent with reef-based diving and snorkelling activities (Fig. 1C and F); corals at the unused platform remained undamaged. In February, lesions associated with the damaged corals recovered, and WS lesions ceased progressing in all but one colony (at the unused platform site). By March, lesions on all WS-affected colonies had healed, although areas of partial colony mortality remained. In June, all colonies were visually healthy.

### Temporal changes in the immune system of corals at the control site

**Figure 2:**
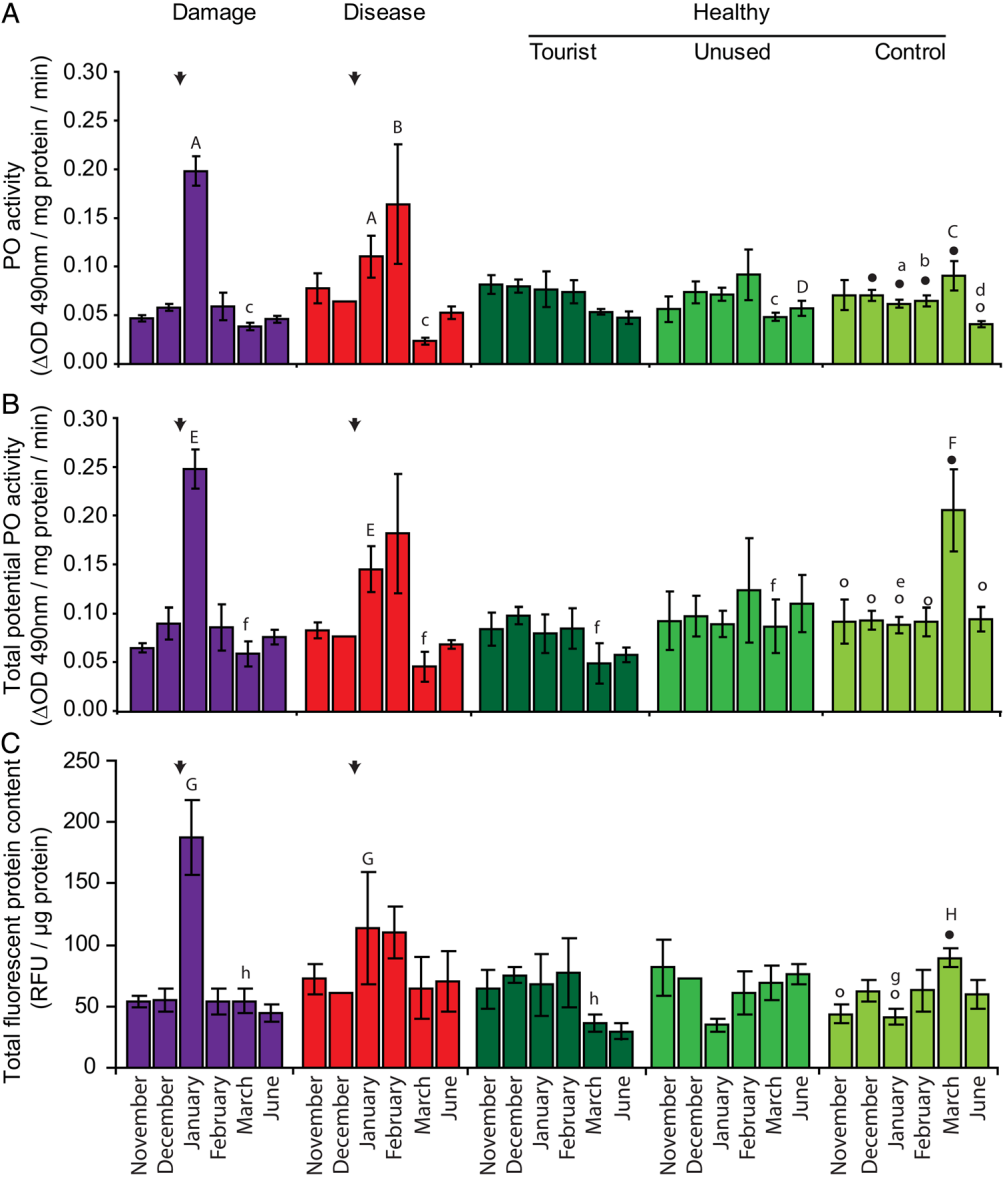
Comparative temporal patterns in phenoloxidase activities and total fluorescence levels in *A. millepora* at Hardy Reef, central Great Barrier Reef*.* Patterns are compared among corals that were healthy at three study sites (tourist platform, unused platform and control site) and those that were damaged or diseased at platform sites, for phenoloxidase (PO; **A**) and total potential phenoloxidase (tpPO) activity (**B**), and total fluorescence levels (**C**). Data are grouped by health status, with healthy corals split up by study location. Arrows indicate when disease and damage occurred. Letters indicate means that differ significantly from the corresponding mean at the control site, where upper case letters (A–H) denote the significantly higher mean in the comparison and lower case letters (a–h) denote the significantly lower mean. For temporal patterns in control corals, symbols (filled or open circles) denote means that differ significantly from means with the other symbol. Results were considered significant when *P* < 0.05 or 95% confidence interval excluded zero.

**Figure 3:**
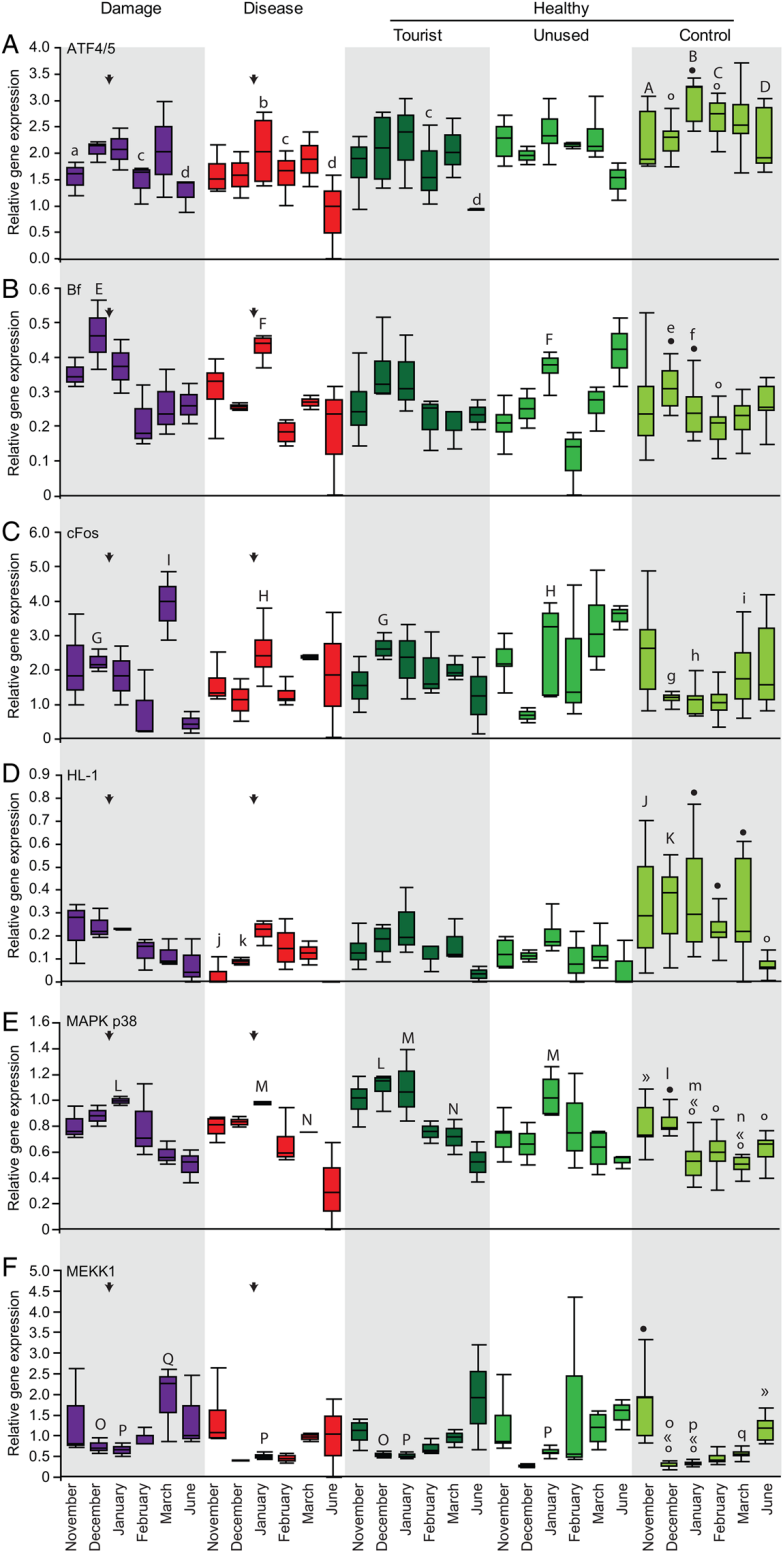
Comparative temporal patterns in immune gene expression levels in *A. millepora* at Hardy Reef, central Great Barrier Reef*.* Patterns are compared among corals that were healthy at three study sites (tourist platform, unused platform and control site) and those that were damaged or diseased at platform sites, for *ATF4/5* (**A**), *Bf* (**B**), *cFos* (**C**), *HL-1* (**D**), *MAPK p38* (**E**) and *MEKK1* (**F**). Data are grouped by health status, with healthy corals split up by study location. Arrows indicate when disease and damage occurred. Letters indicate means that differ significantly from the corresponding mean at the control site, where upper case letters (A–Q) denote the significantly higher mean in the comparison and lower case letters (a–q) denote the significantly lower mean. For temporal patterns in control corals, symbols (filled and open circles, or « and ») denote means that differ significantly from means with the other symbol. Results were considered significant when *P* < 0.05 or 95% confidence interval excluded zero.

Although corals at the control site all remained healthy, expression levels of most immune parameters fluctuated significantly over the 7 months of the study. Four main patterns in the immune response of these healthy control corals were detected. First, mean PO and tpPO activity and total FP concentration (and all component FP classes, i.e. CFP, GFP and RFP) increased in March compared with levels in the previous summer months, although this increase was not significant for PO activity (Fig. 2A–C; Supplementary Fig. S2A–C). Second, we observed increased expression in the complement system’s *Factor B* (*Bf* ) gene in summer months (Fig. 3B) and a similar trend for *C3* (Supplementary Fig. S3B; November to February, *P* = 0.0652), as well as *ATF4/5*, which is involved in the TLR pathway (Fig. 3A). The third pattern observed was that various genes involved in the TLR signalling pathway were expressed at lower levels in summer compared with other months, including *MEKK1* and *MAPK p38* (Fig. 3E and F), while similar trends were found for *cFos* (Fig. 3C; November to December, *P* = 0.0816; November to January, *P* = 0.0652; November to February, *P* = 0.0594) and *TRAF6* (Supplementary Fig. S3K; November > December, *P* = 0.0697). Finally, several parameters showed a pattern of reduced expression or activity in winter (June) compared with all other months. These parameters were PO activity and *HL-1* and *NF-κB* expression (Figs 2A and 3D; Supplementary Fig. S3I). All other parameters showed some temporal fluctuations in expression, but no clear patterns could be discerned. Details of the statistical significance of each comparison can be found in Supplementary File S1 and Supplementary Table S2.

### The effect of anthropogenic disturbances associated with reef platforms on the immune system of corals

In general, temporal patterns in the immune parameters analysed for healthy corals near reef platforms followed profiles similar to those of healthy corals at the control site. In March, however, tpPO activity was reduced in corals at both platform sites in comparison to control corals (Fig. 2B). Total FP tissue concentrations were reduced only in corals at the tourist platform (Fig. 2C), which was attributed to reduced GFP and RFP expression at this site, although RFP expression was also reduced at the unused platform in March. In June, we observed an increase in PO activity at the unused platform (Fig. 2A), while the expression of GFP was reduced at the tourist platform (Supplementary Fig. S2B). Corals near platforms showed significant increases in the expression of genes involved in the TLR signalling pathway in summer. *MEKK1* and *MAPK p38* were upregulated at both platform sites (Fig. 3E and F), as well as *Bf* at the unused platform in January (Fig. 3B). In addition, these genes and the transcription factors *cFos* and *cJun* were upregulated at the tourist platform in December (Fig. 3C; Supplementary Fig. S3C), while these corals had lower *ATF4/5* expression (Fig. 3A) and total fluorescence levels (Fig. 2C) in February and March, respectively.

The putative Toll-like receptor *TIR-1* (Supplementary Fig. S3J) was present in a relatively small portion of the samples regardless of location and month of sampling. One exception occurred at the tourist platform, however, where all healthy corals expressed *TIR-1* at detectable levels during the summer months of January and February. In addition, we found a significant difference in the number of corals with detectable levels of the C-type lectin *CTL-2* between sites (*P* = 0.03; (Supplementary Fig. S3E). This effect was due to the presence of *CTL-2* in 67.5 and 58.2% of the coral samples collected at the control site and at the tourist platform, respectively, while only 35.3% of the samples collected at the unused platform expressed *CTL-2* at detectable levels.

### The immune response of injured corals

In damaged corals, several immune parameters were significantly higher in January, the month when corals sustained injury. Both PO and tpPO activity were increased in injured corals relative to corals at the control site (Fig. 2A and B), as well as total relative fluorescent protein levels, due to increases in CFP, GFP and RFP expression (Fig. 2C; Supplementary Fig. S2A–C) and CP expression (Supplementary Fig. S2D). However, these parameters, except CFP and CP expression (Fig. 2A–C; Supplementary Fig. S2B and C), along with *apextrin* (Supplementary Fig. S3A), were all reduced in March, while *TRAF6*, *MEKK1* and *cFos* were upregulated compared with controls (Supplementary Fig. S2K; Fig. 3C and E). The expression of GFP was also significantly reduced in June (Supplementary Fig. S2B). Signalling via the TLR pathway may also have been involved in the response, with upregulation of *MEKK1* and *MAPK p38* in January (Fig. 3E and F). In addition, elevated expression of the lectin *CTL-1* was observed following physical damage in January (*P* = 0.04; Supplementary Fig. S3D). Surprisingly, we also found increased expression of *Bf*, *cFos*, and *MEKK1* in December, prior to injury (Fig. 3B–E). *ATF4/5* expression was downregulated in both January and February (Fig. 3A).

### The immune response of white syndrome-affected corals

The immune system of corals that developed visual signs of disease showed significantly reduced expression of the lectin *HL-1* and complement gene *C3* prior to January, when signs of WS became apparent (Fig. 3D; Supplementary Fig. S3B). In January, PO and tpPO activities, as well as fluorescent protein levels (in particular CFP and GFP; Supplementary Fig. S2A and B), were increased relative to controls (Fig 2A–C), while expression levels of *Bf*, *cFos*, *MEKK1* and *MAPK p38* were upregulated (Fig. 3B, C, E and F). However, only PO activity was elevated in February (Fig. 2A), while *ATF4/5* expression was downregulated in both January and February (Fig. 3A). In comparison with control site corals, various parameters were differentially expressed or activated in March, including reduced PO and tpPO activities (Fig. 2A and B), as well as *apextrin* levels and upregulation of *ERK-2* (Supplementary Fig. S3A and F).

Corals that showed macroscopic signs of disease had significantly higher levels of *TIR-1* expression (*P* < 0.01; Supplementary Fig. S3J). However, it should be noted that, of all diseased corals analysed in this study (*n* = 4), two consistently expressed *TIR-1* at high levels in all 6 months (average relative expression 5.6 ± 0.88), in contrast to the other two colonies, which had low *TIR-1* expression throughout the study (average relative expression 0.05 ± 0.035).

### The C-type lectin, millectin

*Millectin* was found in the majority of samples, although there was a significant time effect (*P* = 0.01). This was probably due to the absence of expression in 31.6 and 30% of the samples from January and February, respectively; although in contrast, 4.3, 16.7 and 0% of corals lacked expression in November, December and March, respectively (Supplementary Fig. S3H).

Additional details listing the statistical significance of comparisons between corals at the control site and the following: (i) healthy corals near reef platforms; (ii) corals that sustained injury in January; and (iii) corals that developed visual signs of WS in January can be found in Supplementary Table S3.

## Discussion

The presence of 15-fold higher levels of disease as well as injury associated with recreational activities at sites near permanently moored offshore platforms (Lamb and Willis, 2011) provided an important opportunity to compare immune responses of healthy corals with those of corals exposed to a number of anthropogenic and environmental stressors. The immune systems of all corals near reef platforms, including healthy corals, responded to platform-associated stressors, and corals further boosted their immune system in cases of disease or injury over the 7 month sampling period spanning the austral summer. The range of immune responses demonstrated by the coral *A. millepora* in this study highlights the complexity of the coral innate immune system, and such studies enhance understanding of the underlying mechanisms that are contributing to rising levels of coral disease globally (Willis *et al.*, 2004; Sokolow, 2009; Ruiz-Moreno *et al*., 2012).

### Temporal patterns in the immune system of healthy corals

Variation in the expression of immune genes and proteins in healthy control colonies of *A. millepora* suggests that healthy immune systems respond to seasonal variation in environmental parameters, notably in summer. Although no visual signs of disease were detected at the control site, four distinct temporal patterns in immune parameters were detected.

First, upregulation of several genes in summer (significant for *ATF4/5* and *Bf*; trend for *C3*) suggests that visually healthy colonies of *A. millepora* boost components of their immune system in response to summer-related stressors, potentially including increased seawater temperature and/or solar radiation, or reduced salinity associated with the summer wet season. AP-1 transcription factors, such as ATFs, may regulate expression of complement C3, whose gene promoter contains binding sites for these transcription factors. In addition, C3 is activated by Bf in response to the detection of a microbe that requires elimination. Overall, this suggests that the coral complement system is under control of AP-1 transcription factors. Although ATF activity may be regulated by the kinase ERK-2, which itself is activated by stimuli like TLRs and pro-inflammatory cytokine signalling and is known to regulate *C3* expression (Sugihara *et al.*, 2010), our results cannot conclusively confirm the involvement of this pathway in corals. Development of proteomics approaches that investigate the activation status of proteins will undoubtedly reveal additional roles of signalling pathways in coral stress responses. Upregulation of several components of the complement system, potentially in an ERK-2-mediated manner, in summer is consistent with the presence of a seasonally related environmental stressor.

A second pattern detected in healthy control corals provides further support for our interpretation that corals boost their immune system in response to seasonally related environmental stressors. Sudden increases of up to 2.2-fold in both PO and tpPO activities, as well as 1.7-fold increases in total fluorescence levels (due to a rise in CFP, GFP and RFP expression) in late summer (March) strongly suggest that corals experienced a stressor at this time (Fig. 2). However, no anomalies were apparent in seawater temperature, light, rainfall or wind speed data at the sites in March (Supplementary Fig. S1) and, macroscopically, all corals remained visually healthy. Although we can only speculate, the occurrence of peaks in PO activities at the end of summer may represent a response to 3 months of accumulated summer heat stress (Fig. 1C; Supplementary Fig. S1C). Co-occurrence of peaks in PO and tpPO activity with the warmest summer month in a separate study of temporal patterns in the proPO-activating system at Orpheus Island (J. A. J. M. van de Water, unpublished data) lends support for the interpretation that warm summer temperatures are driving this response. Further studies of seasonal patterns in basal levels of immune parameters among populations on outer reefs, such as Hardy Reef, and inshore reefs are needed to test the generality of these patterns. They also highlight the need to investigate further the ecological immunity and basal immune status of corals on larger temporal and spatial scales.

A third pattern detected in healthy control corals was the reduced expression of four genes involved in the TLR signalling pathway (*TRAF6*, *MEKK1*, *MAPK p38* and *cFos*) in summer. This arm of the *TRAF6*-mediated TLR pathway is crucial in immune responses against microbes and may be involved in AMP-mediated regulation of healthy coral-associated microbial communities, such as in *Hydra* (Fraune and Bosch, 2007; Fraune *et al.*, 2010; Franzenburg *et al.*, 2012, 2013). In addition, this signalling cascade is involved in the transcriptional regulation of immune genes, such as immunostimulatory cytokines. We also detected reduced expression of the lectin Millectin in summer, suggesting a compromised lectin–complement pathway and, possibly, a breakdown of the coral–*Symbiodinium* symbiosis, given the role of Millectin in the maintenance of this symbiosis (Kvennefors *et al*., 2008). Reduced function of these immune mechanisms may lead to a less resilient coral. The reduced expression levels found in summer in this study may also help to explain reported increases in disease prevalence in summer (Willis *et al*., 2004; Bruno *et al*., 2007; Harvell *et al*., 2007).

Finally, significant reductions in various immune parameters, including PO activity and the expression of *HL-1* and *NF-κB* in winter (June), are consistent with a seasonally reduced need for these immune parameters. As these are primarily immune effector molecules, this may suggest that corals are exposed to significantly lower levels of microbial stress in winter, which would be consistent with previous observations of shifts in microbial communities (Bourne *et al*., 2008; Mouchka *et al*., 2010; Littman *et al*., 2011; Witt *et al*., 2011) and increased coral pathogen virulence (Sussman *et al*., 2008; Vidal-Dupiol *et al*., 2011) and coral disease (Willis *et al*., 2004; Bruno *et al*., 2007) when seawater temperatures are elevated. This would enable the coral to allocate fewer resources to the immune system and more to other life-history traits, such as growth and reproduction.

### The effect of tourist reef platforms on coral immunocompetence

Significantly higher levels of TLR signalling pathway genes (*MAPK p38* and *MEKK1*) in all corals (healthy, diseased and injured) near both platforms in January, as well as at the tourist platform in December (*MAPK p38*, *MEKK1*, *cFos* and *cJun*), in comparison to control corals, indicates that offshore platforms have an impact on the coral immune system. While physical damage was likely to be the result of reef-based activities, such as snorkelling (Lamb *et al*., 2014), consistent upregulation of this pathway suggests the presence of additional platform-associated stressors, which may have played a role in the development of WS. Potential platform-associated stressors include nutrient influxes derived from guano from sea birds that frequent these reef platforms (Bosman and Hockey, 1986; Bruno *et al.*, 2003), as well as chemicals originating from human activities, such as cleaning products and sunscreen (Blais *et al.*, 2005; Danovaro *et al.*, 2008). Given that the TLR pathway may be involved in regulating the composition of coral-associated bacterial communities, the upregulation of various genes involved in the TLR pathway in corals near platforms in summer could indicate that these corals were responding to changes within their bacterial communities and attempting to re-establish a more favourable community via TLR-induced AMP production. While no cause can be pinpointed, there is a clear need to investigate further the potential anthropogenic disturbances near offshore reef platforms to enable development of management actions that could mitigate the effects of these stressors and prevent localized coral reef degradation.

### The coral response to injury

The primary response to injury detected in this study was up to 3.2-fold increases in the proPO-activating system and up to 5-fold increases in fluorescent protein expression levels in January, when broken branches were first detected. Despite potentially additional, unidentified stressors at platform sites, corals near the tourist platform elicited a sufficient immune response following physical damage to enable recovery. While PO activity may have an antimicrobial function or form a physical barrier to seal the lesion (Mydlarz *et al*., 2008; Palmer *et al*., 2011a, c), GFP-like proteins probably protect regenerating tissues from light stress by dissipating high-energy-wavelength light (Baird *et al.*, 2009). Activation of the melanization cascade and increased levels of GFP-like proteins in tissues at growth margins following physical damage found in an earlier study (D’Angelo *et al.*, 2012; van de Water *et al.*, 2015b) support this interpretation. The GFP-like proteins also have the capacity to scavenge reactive oxygen (Bou-Abdallah *et al.*, 2006; Palmer *et al.*, 2009) and may neutralize radicals produced by the melanization cascade; however, whether this is one of their primary functions is currently unclear. Furthermore, we found that the C-type lectin *CTL-1* was upregulated following damage, suggesting activation of the lectin–complement pathway, which is consistent with studies that show wounding-induced upregulation in the expression of various lectins (Kvennefors *et al*., 2010; van de Water *et al*., 2015b). Another component of this pathway, *Bf*, was also upregulated, although this was observed a month prior to the significant damage event in January. While we have currently no functional explanation for the increased levels of *Bf* in corals that would sustain significant damage later on, it cannot be excluded that these corals were more prone to pressure from reef-based tourist activities, for example based on their proximity to the platform, and responded accordingly. Surprisingly, we did not observe a response via the TLR pathway following physical damage (as previously observed by van de Water *et al*., 2015b). However, the immune response following injury is very dynamic, and we may have missed the fully orchestrated response involving the complement and TLR pathways, which are typically upregulated within days (van de Water *et al*., 2015b), because of low sampling resolution.

### Response of corals to white syndromes

Only corals near reef platforms developed signs consistent with the group of coral diseases known as white syndromes. When combined with the observation that lesions appeared during the warm summer month of January, our results suggest that seasonally related stressors, such as temperature, solar radiation or rainfall, were acting to compound local stressors associated with platforms and cause disease. For example, rainfall events prior to our January 2011 sampling time point may have caused a major influx of nutrients and bacteria associated with bird guano from the platforms onto the reef, thereby potentially affecting the microbial and physiological processes within the coral holobiont, contributing to disease development. Surprisingly, most instances of disease were observed on corals near the unused platform, which had significantly higher levels of bird guano than the tourist platform. While high bird guano influx into the ocean is a potential factor contributing to coral disease, other (still unknown) factors, such as cleaning or antifouling agents used on tourist platforms, cannot be excluded, and investigations into which factors are mainly responsible for disease development are warranted.

Interestingly, we found that prior to disease onset, these corals had significantly reduced levels of the lectin *HL-1* and complement *C3*, suggesting that they may have been immunocompromised, which would have made them more susceptible to disease. However, these differences may also be attributed to biological variation on the genetic or epigenetic level within the coral population. As progression of WS lesions had ceased in all diseased corals by March, we conclude that the immune response was sufficient to halt the disease. The 1.8- to 2.5-fold upregulation of PO activity in January and February, respectively, indicates that the immune response to disease involved activation of the melanization cascade, as well as increased fluorescent protein expression, which was upregulated up to 3-fold compared with control corals in January. In addition, we observed an increase in the expression of the putative TLR, *TIR-1*. While this could indicate that these WS-affected corals regulated additional immune mechanisms in a TLR-dependent manner, it should be noted that expression was highly variable among colonies, and these results should therefore be interpreted with caution.

### Conclusions

Our study of the immune responses of the model coral *A. millepora* to a range of anthropogenic and environmental disturbances reveals that corals have a complex array of immune responses that are differentially regulated according to the type of disturbance. Overall, in corals at the undisturbed control location, expression of a range of immune genes was significantly modulated by seasonal environmental factors, in particular during the summer months. In addition, corals near tourist platforms showed upregulation of genes involved in the TLR signalling pathway in summer, suggesting increased pressure on the coral immune system from reef platform-associated factors. As a result, the coral immune system may have been overwhelmed by the combined or synergistic effects of these stressors, leading to disease in corals near platforms. However, corals that developed disease or sustained injury responded effectively, primarily using the melanization cascade and enhanced GFP-like protein expression, and recovered when summer heat stress subsided. Further whole-transcriptome or proteome analyses may provide a more detailed understanding of the impacts that local anthropogenic stressors have on physiological and stress response processes in corals. Overall, our study shows that corals are able to cope with normal seasonal stressors in summer, but that additional anthropogenic stressors compromise their immune system, contributing to increased disease prevalence and thereby localized reef degradation at these sites. Identifying the anthropogenic stressors responsible will enable the implementation of management actions to reduce stress on corals and will aid conservation efforts.

## Supplementary material

Supplementary material is available at *Conservation Physiology* online.

## Funding

This work was supported by the Australian Research Council through funds allocated by the Centre of Excellence for Coral Reef Studies to B.L.W. (ARC CEO561435), and by AIMS@JCU through a grant awarded to J.B.L.

## Supplementary Material

Supplementary Data
