# Supplementary material for: Comparative immune responses of corals to stressors associated with offshore reef-based tourist platforms
Source: Conserv Physiol. 2015 Jul 24;3(1):cov032. doi: 10.1093/conphys/cov032 (PMC4778433; doi:10.1093/conphys/cov032)
Supplement: Supplementary Data [file cov032supp.zip › cov032supp.docx]

**Supplementary tables**

**Supplementary table 1 – List of immune genes**

| **Gene name** | **Acronym** | **Accession number** | **PCR product length** | **Sequence of primer coding region** | **References** |
| --- | --- | --- | --- | --- | --- |
| **Reference gene** |  |  |  |  |  |
| Glyceraldehyde 3 phosphate dehydrogenase | *GAPDH* | EZ026309 | 187 | F: AGGTGGAGCCAAGAAAGTCA  R: TTAGCTAGAGGAGCCAGGCA | (Csaszar et al., 2009; Seneca et al., 2010) |
| Ribosomal protein S7 | *RPS7* | EZ031290 | 197 | F: CAGGCATGCTTACAACCAAA  R: TCAACCTCCTTTGCTCCAGT |  |
| Ribosomal protein L9 | *RPL9* | EZ026324 | 292 | F: CGTGTAAACGTGTGGTTTGC  R: TTTGACACCTGAATTCGCAC |  |
| Unknown transcript | *Ctg_1913* | EZ040581 | 280 | F: GATTTAACCACCGGCAGTGT  R: ATGGTAGGGAGGAGGCTGTT |  |
| **Toll-like receptor pathway** |  |  |  |  |  |
| Toll interleukin receptor | *TIR-1* | EF090256 | 137 | F: AAAGCCGCAGTCATCAGTTT  R: GAAATTGGCGTTGAATTCGT | (Miller et al., 2007) |
| TNF receptor associated factor 6 | *TRAF-6* | DY583189 | 127 | F: TGATGAATGTCCTTTCGCAG  R: ACATGCTTTGCAAGCTGATG |  |
| Mitogen-activated protein kinase/ERK kinase kinase-1 | *MEKK-1* | DY581208 DY581138  DY582675 | 117 | F: CTGCGGATATTTGGTCCTGT  R: TTTCTTTGTCGGTTGATCCC |  |
| Extracellular signal regulated kinase | *ERK-2* | EZ025389 | 217 | F: CCAAAGGTTACAGCAAGGCT  R: TGCGTGCCTTTTCATTCATA |  |
| Mitogen-activated protein kinase p38 | *MAPK p38* | EZ031759 | 237 | F: AAAATCAGCAGTGAATCCGC  R: TCGGGGTCTGAATACGTAGC |  |
| Component of AP-1 transcription factor | *cFos* | EZ016042 | 177 | F: CTGGAAAGAGAATTGCTGGC  R: GACGATTGCACTTCGGACTT |  |
| Component of AP-1 transcription factor | *cJun* | EZ020860 | 366 | F: TCGATCGAAGGGACAGTTCT  R: GTGCTAGTTGCGGTGTTCAA |  |
| Activating transcription factor 4 | *ATF4/5* | DY577805 | 247 | F: GGCCAGAACGTATCACCAAT  R: TCTTCGAAATCAAACCCCTG |  |
| Nuclear factor kappa B | *NF-kB* | G0000491  G0002043  DY582971  DY580118 | 152 | R: CTCATATGCAGGTTGGTGGA  F: GATGTTGCAGGCTCAGTTCA |  |
| TRIF-related adaptor molecule | *TRAM* | EZ047194 | 157 | F: AAGCTAACGGCTCACCAAGA  R: TGTGCCATGCACAAGAAAAT |  |
| **Alternative complement pathway** |  |  |  |  |  |
| Complement factor B | *Bf* | GO001635 | 227 | F: TTATCCATCCCGACGCTAAC  R: AGGATCATCTTTTCCTGCGA | (Kimura et al., 2009) |
| Complement C3 | *C3* | EF090257 | 167 | F: CCGCTACACGCTAGACAACA  R: CCGCAGAGTCGATGTACAAA |  |
| **Lectins** |  |  |  |  |  |
| Mannose-binding lectin | *millectin* | EU717895 | 257 | F: AGCGAGTATCCACAACACCC  R: GGCTTTTTCGATGTTTTCCA | (Kvennefors et al., 2008) |
| C type lectin-1 | *CTL-1* | GO001638 | 312 | F: GGGTTGTGTACAACGGCTTT  R: CTTTCCATTCGGTTCTCCTG | (Grasso et al., 2008; Grasso et al., 2011) |
| C type lectin-2 | *CTL-2* | GS01UH10 | 267 | F: CAGGTCTGGATCGGACTCAT  R: CATGTCCAGTGGTTGTACGC |  |
| Hemolytic lectin-1 | *HL-1* | EU863776  EU863777 | 335 | F: TTCGCTCCAGAGGGAAACTA  R: GCAGAAATGCCTTTGGTTGT | (Grasso et al., 2008; Grasso et al., 2011) |
| Hemolytic lectin-2 | *HL-2* | EU863776 | 302 | F: AACAGTTGAGATAACCGCCG  R: TTGATTCCTGGTGCATTTGA |  |
| Hemolytic lectin-3 | *HL-3* | EU863777 | 379 | F: TTCTGGAGATTGGGTAACGC  R: TCGTTCTCAGCGTGTTGTTC |  |
| **Membrane attack complex / perforin** |  |  |  |  |  |
| Apical extracellular protein | *Apextrin* | EF091848 | 352 | F: GGATTCGTACCAAAAAGGCA  R: GAGGGGTCTGATATGGGGTT | (Miller et al., 2007) |
| 60 kDa proteinaceous toxin | *Tx60-A* | DY579588 | 207 | F: TACTGCCCTTGAGGTTTGCT  R: CTGAAAATCCCGCTGACTGT |  |

At the 5’-end, all primers contained universal fluorescent tags. Forward primer: AGGTGACACTATAGAATA. Reverse primer: GTACGACTCACTATAGGGA.

**Supplementary table 2** – Overview of pair-wise comparisons between means of immune parameters measured in corals at the control site. Only statistically significant comparisons are shown. Results were considered significant when p < 0.05 after applying the Tukey’s Honest Significant Differences multiple comparison correction.

|  | **p-value** |  | **p-value** |  | **p-value** |
| --- | --- | --- | --- | --- | --- |
| ***PO activity*** |  | ***GFP*** |  | ***MAPK p38*** |  |
| December – June | <0.001 | January – March | 0.0471 | November – January | 0.0378 |
| January – June | <0.001 | ***RFP*** |  | November – March | <0.001 |
| February – June | <0.001 | November – March | <0.001 | December – January | <0.001 |
| March – June | 0.0045 | January – March | <0.001 | December – February | 0.0252 |
| ***tpPO activity*** |  | February – March | <0.001 | December – March | <0.001 |
| November – March | 0.0010 | ***Chromoprotein*** |  | December – June | 0.0397 |
| December – March | 0.0012 | November – June | 0.0161 | ***MEKK1*** |  |
| January – March | 0.0006 | ***ATF4/5*** |  | November – December | 0.0354 |
| February – March | 0.0010 | December – January | 0.005 | November – January | 0.0408 |
| March – June | 0.0015 | January – February | <0.001 | December – June | <0.001 |
| ***Total*** ***fluorescence*** |  | ***Bf*** |  | January – June | <0.001 |
| November – March | <0.001 | December – February | <0.001 | February – June | <0.001 |
| January – March | <0.001 | January – February | <0.001 | ***NFκB*** |  |
| ***CFP*** |  | ***HL1*** |  | November – June | <0.001 |
| November – March | 0.0087 | January – June | 0.0046 | December – June | 0.0246 |
| January – March | 0.0426 | February – June | <0.001 | January – June | 0.0452 |
|  |  | March – June | 0.0118 | February – June | 0.0101 |

**Supplementary table 3** – Overview of pair-wise comparisons of immune parameters between corals at the control site and corals that either 1) remained healthy at platform sites (Tourist Healthy and Unused Healthy), 2) developed disease in January (Disease), or 3) sustained physical damage in January (Damage). Only statistically significant comparisons are shown. Results were considered significant when 95% confidence intervals excluded 0.

|  | **Comparison** | **95% Confidence Interval** | |  | **Comparison** | **95% Confidence Interval** | |
| --- | --- | --- | --- | --- | --- | --- | --- |
| ***PO activity*** |  |  |  | ***ATF4/5*** |  |  |  |
| January | Damage | 0.0992 | 0.1730 | November | Damage | -1.460 | -0.0024 |
|  | Disease | 0.0148 | 0.0818 | January | Disease | -1.69 | -0.1570 |
| February | Disease | 0.0256 | 0.1730 | February | Damage | -1.86 | -0.537 |
| March | Damage | -0.0905 | -0.0134 |  | Disease | -1.67 | -0.469 |
|  | Disease | -0.1120 | -0.0222 |  | Tourist | -1.62 | -0.298 |
|  | Unused | -0.0749 | -0.0095 | June | Damage | -1.89 | -0.103 |
| June | Unused | 0.00140 | 0.0311 |  | Disease | -2.29 | -0.505 |
| ***tpPO activity*** |  |  |  |  | Tourist | -2.35 | -0.302 |
| January | Damage | 0.1120 | 0.2070 | ***C3*** |  |  |  |
|  | Disease | 0.0135 | 0.0996 | December | Disease | -1.920 | -0.196 |
| March | Damage | -0.264 | -0.0317 | ***cFos*** |  |  |  |
|  | Disease | -0.296 | -0.0260 | December | Damage | 0.560 | 1.620 |
|  | Unused | -0.218 | -0.0212 |  | Tourist | 1.010 | 1.970 |
|  | Tourist | -0.274 | -0.0414 | January | Disease | 0.148 | 2.71 |
| ***Fluorescence*** |  |  |  |  | Unused | 0.358 | 2.75 |
| January | Damage | 79.4 | 210.0 | March | Damage | 0.5140 | 3.46 |
|  | Disease | 11.8 | 130.0 | ***cJun*** |  |  |  |
| March | Damage | -69.8 | -0.606 | December | Tourist | 0.0706 | 0.777 |
|  | Tourist | -87.2 | -18.00 | June | Damage | -1.130 | -0.0737 |
| ***Cyan FP*** |  |  |  |  | Disease | -1.090 | -0.0367 |
| January | Damage | 42.4 | 143.0 | ***ERK2*** |  |  |  |
|  | Disease | 2.88 | 94.3 | March | Disease | 0.053 | 1.930 |
| ***Green FP*** |  |  |  | ***HL1*** |  |  |  |
| January | Damage | 12.0 | 38.7 | November | Disease | -0.515 | -0.06160 |
|  | Disease | 2.32 | 26.4 | December | Disease | -0.478 | -0.011 |
| March | Damage | -21.5 | -1.95 | ***MAPK p38*** |  |  |  |
|  | Tourist | -20.7 | -1.16 | December | Tourist | 0.096 | 0.425 |
| June | Damage | -18.5 | -4.89 | January | Damage | 0.183 | 0.746 |
|  | Tourist | -14.8 | -2.52 |  | Disease | 0.225 | 0.666 |
| ***Red FP*** |  |  |  |  | Tourist | 0.324 | 0.808 |
| January | Damage | 14.0 | 36.8 |  | Unused | 0.307 | 0.718 |
| March | Damage | -23.5 | -8.19 | March | Disease | 0.064 | 0.439 |
|  | Disease | -18.9 | 1.14 |  | Tourist | 0.054 | 0.377 |
|  | Tourist | -23.3 | -8.06 | ***MEKK1*** |  |  |  |
|  | Unused | -15.3 | -2.31 | December | Damage | 0.295 | 0.585 |
| ***Chromoprotein*** |  |  |  |  | Tourist | 0.106 | 0.369 |
| January | Damage | 9.47 | 56.9 | January | Damage | 0.143 | 0.515 |
| ***Apextrin*** |  |  |  |  | Disease | 0.035 | 0.325 |
| March | Damage | -0.299 | -0.0047 |  | Tourist | 0.029 | 0.349 |
|  | Disease | -0.344 | -0.0019 |  | Unused | 0.135 | 0.407 |
| ***Bf*** |  |  |  | February | Unused | 0.071 | 2.52 |
| December | Damage | 0.0299 | 0.2730 | March | Damage | 0.366 | 1.99 |
| January | Disease | 0.07670 | 0.287 |  |  |  |  |
|  | Unused | 0.01560 | 0.212 |  |  |  |  |
