# Supplementary material for: Comparative immune responses of corals to stressors associated with offshore reef-based tourist platforms
Source: Conserv Physiol. 2015 Jul 24;3(1):cov032. doi: 10.1093/conphys/cov032 (PMC4778433; doi:10.1093/conphys/cov032)
Supplement: Supplementary Data [file cov032supp.zip › cov032supp_figs.pdf]

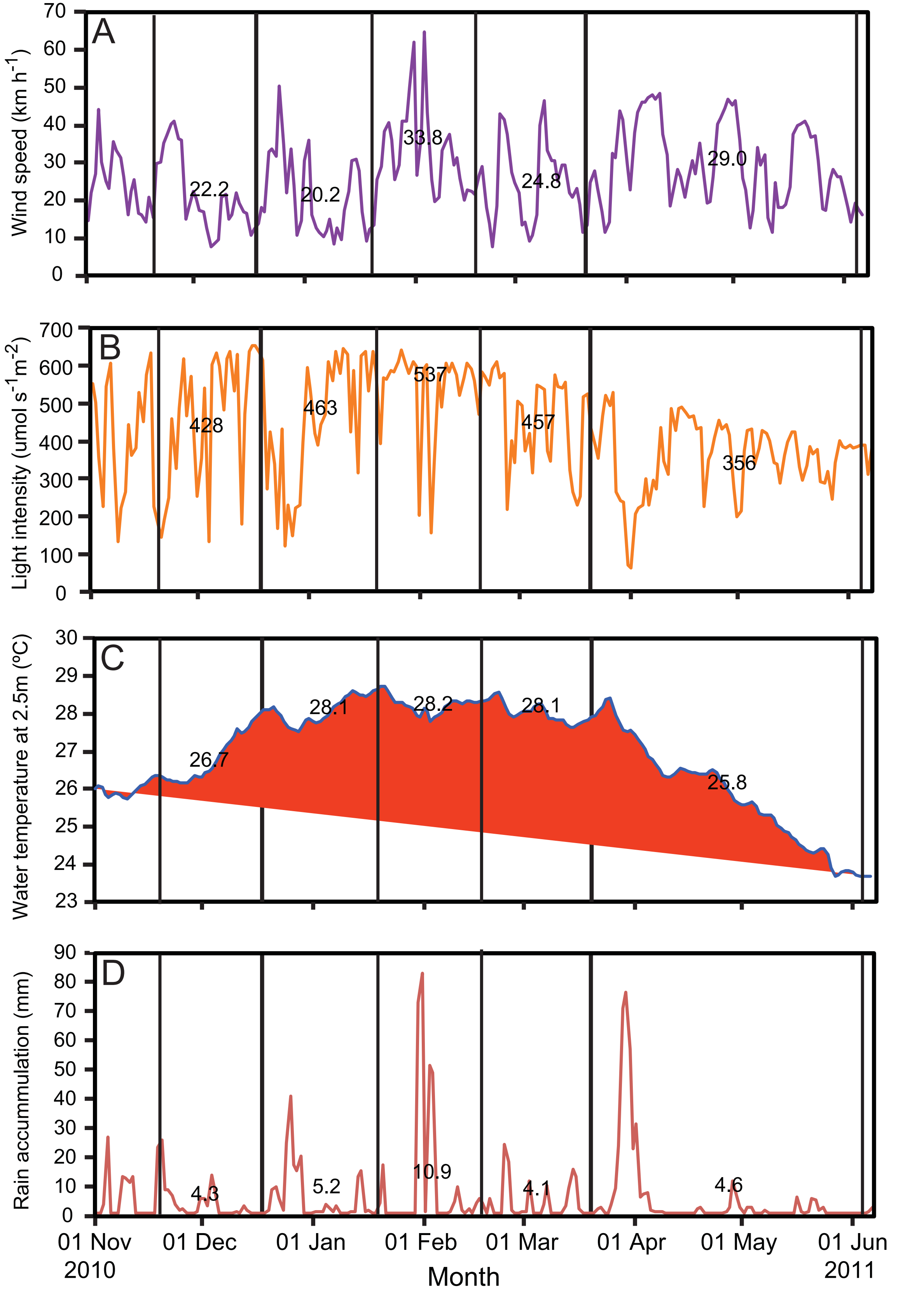

Supplementary figure 1 – Daily means of the environmental factors: A) wind speed (km h-1), (B) light intensity (photosynthetically active radiation, µmol s-1 m-2), (C) water temperature (2.5 m depth, ºC) and (D) rainfall accumulation (mm). Bold vertical lines indicate sampling time points. Numbers between vertical lines represent mean daily values between time points. Environmental data were obtained from the Australian Institute of Marine Science (AIMS) monitoring station located on the main tourist platform at Hardy Reef.

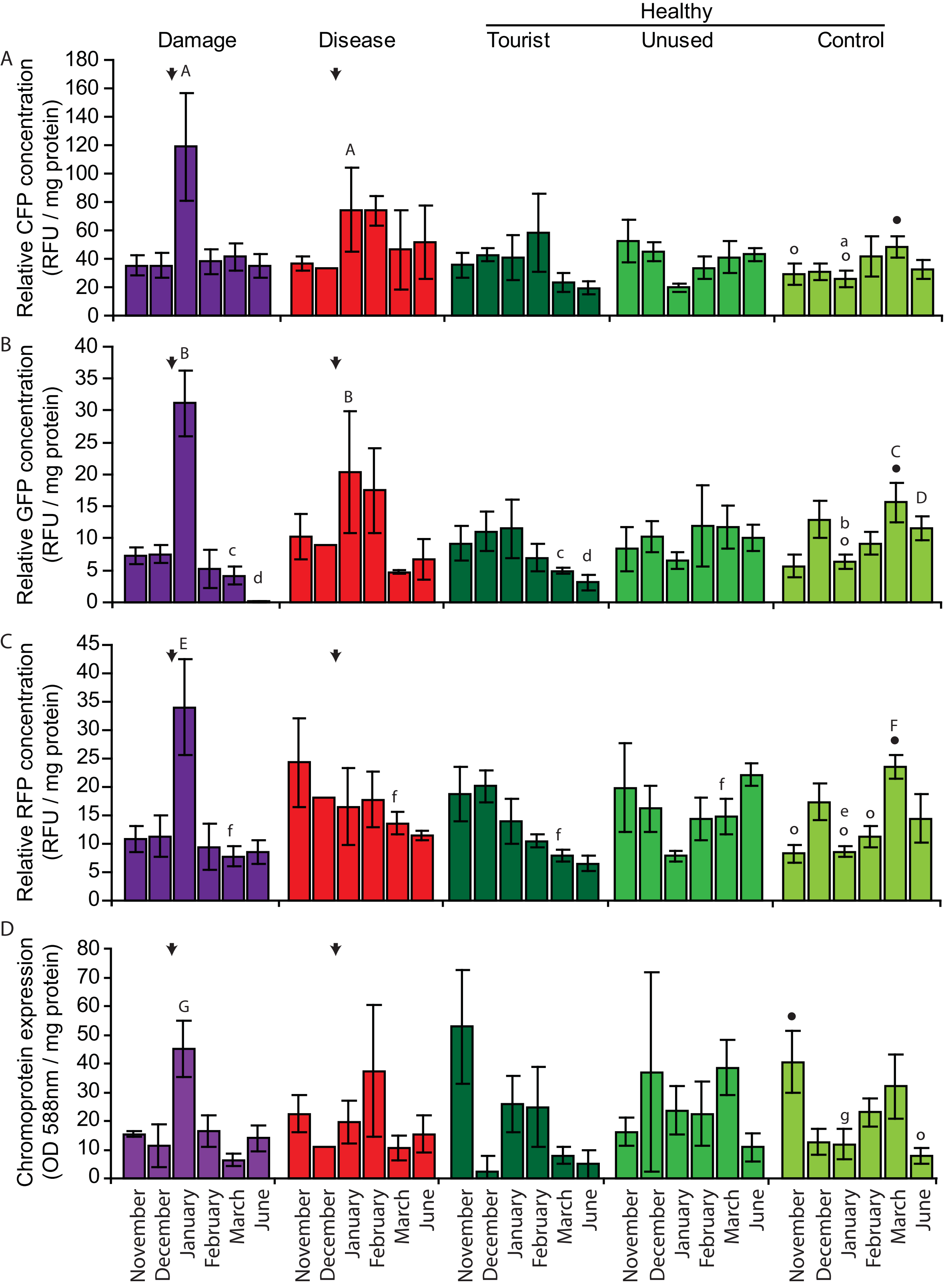

Supplementary figure 2 - Comparative temporal patterns in GFP-like protein expression in *Acropora millepora* at Hardy Reef, central Great Barrier Reef. Patterns are compared among corals that were healthy at three study sites (tourist platform, unused platform and control site), and those that were damaged or diseased at platform sites, for: A) proportion of CFP, B) proportion of GFP, C) proportion of RFP and D) chromoprotein levels. Data are grouped by health status, with healthy corals split up by study location. Arrows indicate when disease and damage occurred. Letters indicate means that differ significantly from the corresponding mean at the control site, where upper case letters (A-C) denote the significantly higher mean in the comparison, and lower case letters (a-c) denote the significantly lower mean. For temporal patterns in control corals, symbols (• or ⊠) denote means that differ significantly from means with the other symbol. Results were considered significant when $p < 0.05$ or 95% confidence interval excluded 0.

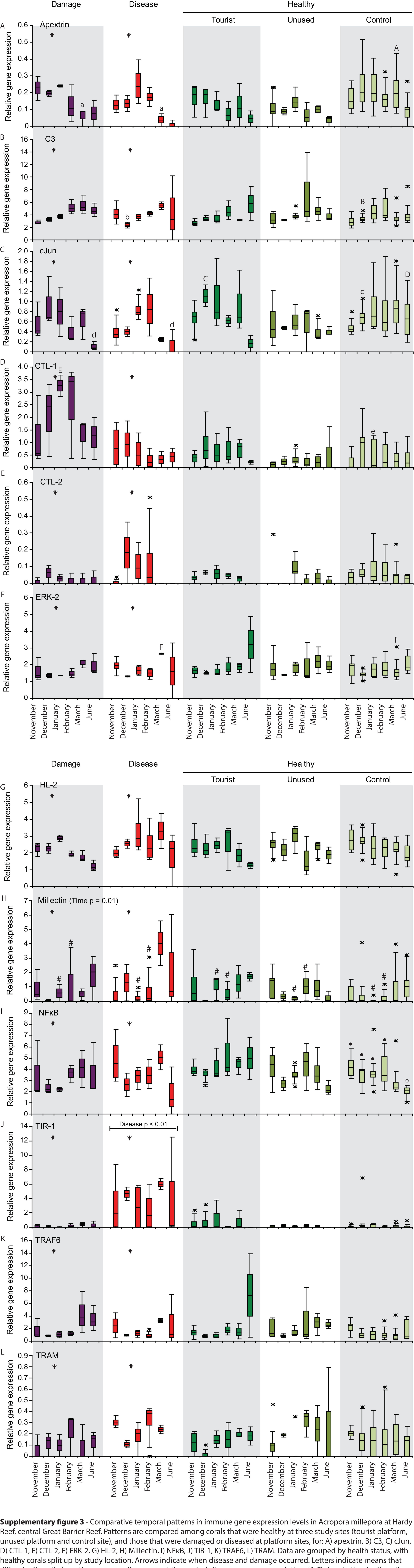

Supplementary figure 3 - Comparative temporal patterns in immune gene expression levels in Acropora millepora at Hardy Reef, central Great Barrier Reef. Patterns are compared among corals that were healthy at three study sites (tourist platform, unused platform and control site), and those that were damaged or diseased at platform sites, for: A) apextrin, B) C3, C) cJun, D) CTL-1, E) CTL-2, F) ERK-2, G) HL-2, H) Millectin, I) NFκB, J) TIR-1, K) TRAF6, L) TRAM. Data are grouped by health status, with healthy corals split up by study location. Arrows indicate when disease and damage occurred. Letters indicate means that differ significantly from the corresponding mean at the control site, where upper case letters (A-F) denote the significantly higher mean in the comparison, and lower case letters (a-f) denote the significantly lower mean. For temporal patterns in control corals, symbols (• or ⊠) denote means that differ significantly from means with the other symbol. Symbol # indicates months where proportion of corals expressing immune gene is significantly lower compared to other months. Results were considered significant when $p < 0.05$ or 95% confidence interval excluded 0.
